# Amygdalar and Hippocampal Morphometry Abnormalities in First-Episode Schizophrenia Using Deformation-Based Shape Analysis

**DOI:** 10.3389/fpsyt.2020.00677

**Published:** 2020-07-17

**Authors:** Xiaoying Tang, Guiwen Lyu, Minhua Chen, Weikai Huang, Yin Lin

**Affiliations:** ^1^ Department of Electrical and Electronic Engineering, Southern University of Science and Technology, Shenzhen, China; ^2^ Department of Radiology, The First Affiliated Hospital of Shenzhen University, Shenzhen, China; ^3^ Department of Electrical and Electronic Engineering, Harbin Institute of Technology Shenzhen Graduate School, Shenzhen, China; ^4^ Department of Psychology, Shenzhen Children's Hospital, Shenzhen, China

**Keywords:** first-episode schizophrenia, amygdala, hippocampus, deformation, shape, subregion

## Abstract

In this study, we investigated and quantified the amygdalar and hippocampal morphometry abnormalities exerted by first-episode schizophrenia using a total of 92 patients and 106 healthy control participants. Magnetic resonance imaging (MRI) based automated segmentation was conducted to obtain the amygdalar and hippocampal segmentations. Disease-versus-control volume differences of the bilateral amygdalas and hippocampi were quantified. In addition, deformation-based statistical shape analysis was employed to quantify the region-specific shape abnormalities of each structure of interest. To better identify the key relevant areas in the pathology of first-episode schizophrenia, each structure was divided into four subregions; CA1, CA2, CA3 combined with dentate gyrus for the hippocampus in each hemisphere and basolateral, basomedial, centromedial, and lateral nucleus for the amygdala in each hemisphere. We observed significant global volume reduction and localized shape atrophy in each of the four structures of interest. The amygdalar shape abnormalities mainly occurred at the basolateral and centromedial subregions, whereas the hippocampal shape abnormalities mainly concentrated on the CA1 and CA2 subregions. For the same structure, the one on the right hemisphere was affected more by the disease pathology than that on the left hemisphere. To conclude, we have successfully quantified the global and local morphometric abnormalities of the bilateral amygdalas and hippocampi using a sophisticated statistical analysis pipeline and high-field subregion segmentations, with MRI data of a considerable sample size. This study is one of the very first of such kind in first-episode schizophrenia analyses.

## Introduction

Schizophrenia is a chronic mental disorder characterized by positive symptoms such as distorted perception and delusional beliefs, negative symptoms such as flattened affect and social withdrawal, as well as cognitive impairments such as deficits in working memory, attention, problem solving, processing speed, and social cognition (1). The first episode of schizophrenia normally occurs in late teens or early twenties. Studying patients at first episode psychosis provides an opportunity to investigate the disease without potential confounding effects of antipsychotic medication or secondary effects such as social deprivation, which may provide a fundamental understanding of the pathological changes in schizophrenia.

Based on structural magnetic resonance imaging (MRI), significant whole brain volume deficits have been identified in first-episode schizophrenia (FES) (2–6), especially those in the frontal lobe, striatum, and limbic system. Two key components of the limbic system are the amygdala and the hippocampus, and the volumes of those two structures have been found to be affected significantly by the neuropathology of FES (4–13). However, findings are controversial and some neuroimaging studies reported conflicting results. For example, some studies reported volumetric reductions of the bilateral amygdalas in FES (3, 8, 9, 13) whereas some other studies reported no significant amygdalar volume changes when comparing FES patients to matched healthy control (HC) participants (6, 12). This type of conflicting findings occur to the hippocampus as well, the structure which has been investigated more extensively than the amygdala in FES literature; some MRI studies observed no significant FES related hippocampal volume abnormalities (8, 10, 12, 13) even though a majority of existing studies have identified hippocampal volume atrophies in FES patients (5–7, 11).

A potential reason of these relatively conflicting findings is that the structure volume is a global measurement and thus it cannot quantify localized morphometrics. It is plausible that there exist very localized morphometric abnormalities which cannot be effectively detected based on the structure volume. Also, it is plausible that different subregions of a single structure have varying localized morphometric phenotype; for example, some parts have localized volume atrophy while some other parts have localized volume expansion, which makes the overall structure volume unaffected.

In such context, mapping based statistical shape analyses, although relatively rare, were employed to characterize and quantify the localized amygdalar and hippocampal morphometric abnormalities in FES (11, 13). The primary limitations of those previous FES related shape analyses are two-fold: Firstly, the sample size was typically very limited [62 FES and 60 controls in one study (11); 28 FES and 28 controls in the other (13)]. Secondly, no subregion definitions were provided for either the amygdala or the hippocampus. Also, the approach employed in Narr et al. (11) analyzed the localized distance to the medial curve of the surface of each structure of interest, the relationship of which to morphometry is relatively implicit. There have also been similar mapping based hippocampal shape analyses focusing on schizophrenia but not FES (14, 15).

In this study, we investigated FES related morphometric abnormalities of the amygdala and the hippocampus in both hemispheres in a coarse-to-fine manner; both global volume and localized shape morphometric abnormalities were quantified based on the structural MR images of 92 FES patients and 106 HCs. A well-established deformation based statistical shape analysis pipeline was employed in the framework of large deformation diffeomorphic metric mapping (LDDMM) (16). This pipeline has been successfully applied to analyzing the shape phenotype of the amygdala and the hippocampus in a variety of brain disorders, such as Alzheimer's disease (16–23), Huntington's disease (24), attention deficit hyperactivity disorder (25), Wilson's disease (26), and other types of brain researches (27–29). Moreover, we investigated the shape abnormalities of those two structures within multiple functionally distinct subregions; CA1, CA2, CA3 combined with dentate gyrus, and subiculum subregions for the hippocampal shape, and basolateral, basomedial, centromedial, and lateral nucleus subregions for the amygdalar shape. Characterizing subregional FES related abnormalities in the bilateral amygdalas and hippocampi may help better elucidate the underlying patho-physiological mechanisms associated with FES and also identify the specific functional systems disturbed in FES.

In view of previous structural MRI findings, we hypothesized amygdalar and hippocampal morphometric abnormalities, and specifically atrophies, in terms of both volume and shape in FES. We also hypothesized that the shape atrophies of those two structures in FES are inhomogeneous, varying from surface vertex to surface vertex, and also from subregion to subregion.

## Material and Methods

### Participants

In this study, the FES patients were recruited from the First Affiliated Hospital of Shenzhen University, from March 2008 to November 2018. Clinical evaluations were conducted by two experienced psychiatrists, with over 5 years of clinical work as attending physicians. Consensus diagnosis of first episode of schizophrenia was determined following the Diagnostic and Statistical Manual of Mental Disorders, Fourth Edition (DSM-IV) Axis I Disorders (SCID-P; patient version). Patients with schizophreniform psychosis were included in the study only if they were found to meet the DSM-IV diagnostic criteria of schizophrenia after being followed up for at least 6 months and at most 12 months. The follow-up was performed by contacting the family members or the patients *via* telephone interviews. If a patient and his/her family could not be reached, or a participant no longer met the criteria of schizophrenia, he/she was excluded from the study. All patients were experiencing first-episode psychosis and were treatment-naïve at the time of clinical assessment and MRI scan.

A total of 106 HCs were recruited from the local community by poster advertisement. They were interviewed by experienced psychiatrists using the SCID non-patient version to ensure the absence of any major mental disorder. Individuals who were pregnant or had any history of alcohol or drug abuse, or any severe neurological illness such as brain tumor or epilepsy were excluded. All participants were Han Chinese and right-handed. Brain MRI images of all participants were inspected by an experienced neuroradiologist and no gross abnormality was observed in any participant. The following exclusion criteria applied to both groups: 1) history of substance abuse or dependence, 2) significant systemic or neurologic illness as assessed by clinical evaluations and medical records, and 3) comorbid affective illness or schizoaffective disorder. This research was approved by the First Affiliated Hospital of Shenzhen University Ethics Committee, and was in accordance with the Declaration of Helsinki. Written informed consent was given by all participants or family relatives after being provided with a complete description of the study.

Demographic characteristics are listed in **Table 1**. A total of 198 samples were obtained, including 92 FES patients (50 females and 42 males) and 106 control participants (47 females and 59 males), aged from 12 to 43 years (FES patients' average age = 20.40 ± 5.59 years; HCs' average age = 23.68 ± 4.04 years). There is no significant group difference at either sex (P = 0.16), age (P = 0.071), or duration of education (P = 0.43). The Global Assessment of Functioning (GAF) (30) and the Positive and Negative Syndrome Scale (PANSS) (1) were used to respectively assess the social function and clinical symptom severity of patients with FES.

**Table 1:** Demographic information for participants included in this study.

Variables	FES patients (n = 92)	Healthy controls (n = 106)
Gender (male/female)	42/50	59/47
Age (years)	22.40 ± 5.59	23.68 ± 4.04
Duration of education (years)	12.32 ± 3.18	12.76 ± 3.29
Age of onset (years)	21.26 ± 5.29	
GAF scores	29.16 ± 10.18	
PANSS scores:		
Total	93.90 ± 16.24	
Positive	24.55 ± 6.31	
Negative	20.89 ± 8.46	
General psychopathology	48.46 ± 8.39	

### MRI Data Acquisition

All structural MRI data were acquired on a Siemens Trio Tim 3T scanner. For each participant, T1-weighted 3D volume image of the whole brain was acquired using a magnetization prepared-rapid acquisition gradient echo (MPRAGE) sequence with the following scanning parameters: repetition time = 13.40 ms, echo time = 4.6 ms, flip angle = 20°, field of view = 256 × 256, and 1-mm^3^ isotropic resolution across the entire cranium. All MR images were visually inspected by one neuroradiologist for data quality control.

### Volumetric Segmentation

We used a validated automatic segmentation algorithm, the multi-atlas likelihood fusion (MALF) (31, 32), for segmenting the bilateral amygdalas and hippocampi from each T1-weighted image. MALF relies on the information of multiple atlases, each of which consists of an MR brain image and a pre-defined segmentation map. In this study, we used 45 atlases, each of which had previously been segmented into a total of 289 anatomical regions, including our four structures of interest (left and right amygdala and hippocampus). The segmentation pipeline and the atlases are freely accessible at the MriCloud platform (www.mricloud.org). All of our segmentation results were visually examined and manually corrected in case of segmentation error or inaccuracy.

### Shape Analysis

After segmenting out the 3D volume of each structure of interest, namely the left and right amygdala and hippocampus, from each T1-weighted image, we created its corresponding 2D contouring surface using a fully-automated surface generation pipeline ensuring sufficient smoothness and correct anatomical topology (33). We then generated a common structure-specific template surface using all surfaces from the 198 participants, utilizing a Bayesian template estimation algorithm (34). After that, the LDDMM-surface algorithm (35) was applied to map the structure-specific template to each participant's surface of the same structure, from which a structure-specific and participant-specific diffeomorphism was obtained. We then extracted the structure-specific and participant-specific deformation marker to be the determinant of the Jacobian of the corresponding diffeomorphism. This deformation marker quantifies the localized morphometrics in the same registered coordinates, for which a positive value indicates surface expansion in the participant relative to the template and a negative value implies the participant's surface atrophy relative to the template.

To better identify the key FES target region of each structure of interest, we performed subregion division using a well-established pipeline (16). For the amygdala in each hemisphere, we divided it into four subregions: basolateral, basomedial, centromedial, and lateral nucleus. For the hippocampus in each hemisphere, we also divided it into four subregions: CA1, CA2, CA3 combined with dentate gyrus (CA3/DG), and subiculum.

### Statistical Analysis

For each structure of interest, let *J_k_*(*s*) denote the deformation marker at vertex *k* of the template surface for participant *s*, we used the following linear model for shape group comparison, namely $J_{k}(s)=\beta_{k,0}+\beta_{k,1}\gamma (s)+\sum_{\mathrm{cov}}\alpha_{\mathrm{cov}}X_{\mathrm{cov}}(s)+\epsilon_{k}(s)$, where *γ*(*s*) is a binary group variable (*γ*(*s*) equals to 1 if participant *s* belongs to FES and 0 otherwise), *X*_cov_(*s*) is the covariate information included in the analysis (in this study, we covaried for age, sex and intracranial volume), and *ϵ_k_*(*s*) denotes a Gaussian noise variable. We tested the null hypothesis that *β_k_*_,1_ = 0 separately for each vertex *k*. Statistics were therefore computed at all vertices, and *p*-values were corrected for multiple comparisons by controlling the family-wise error rate (FWER) at a level of 0.05. Please note the *p*-values were corrected at the vertex-level of each structure of interest. The statistical significance of a difference between FES and HC was quantified *via* Fisher's randomization and permutation tests; we used Monte Carlo simulations to generate 10,000 uniformly distributed random permutations. The degree of vertex-wise shape group difference is represented by *β*_*k*,1_. And thus, negative values denote atrophy (compression) in FES whereas positive values denote expansion when compared to HC.

For volume group comparison, we used the same linear regression model, but there was no need for multiple comparison correction. To note, for each structure, we standardized the volume measurements by z-score transformations; subtracting the average volume of the HC group and dividing it by its standard deviation. It is worthy of being pointed out that we did not adjust multiple testing across the multiple volume analyses nor the multiple shape analyses because our goal was to analyze each single structure of interest and each single morphometric measure (shape and volume).

## Results

### Volume Comparison

The mean values and standard deviations of the volume measurements of the left and right amygdala and hippocampus within FES and HC, as well as the P values and volume differences (in terms of both absolute volume and z-score) obtained from HC-versus-FES group comparisons are listed in **Table 2**. Clearly, for each of the four structures of interest, there was significant volume reduction in the FES group when compared to the HC group. The degrees of volume reductions were similar across structures, with the z-score differences being around −0.3. As revealed by the P values (left amygdala: P < 0.0001, right amygdala: P < 0.0001, left hippocampus: P = 0.0116, right hippocampus: P = 0.0229), the significance of the amgygdalar volume reductions was much stronger than that of the hippocampal volume reductions. Cohen's d is reported as a measure of effect size for the volumetric group differences, which are respective 0.419, 0.541, 0.331, and 0.263 for the left amygdala, the right amygdala, the left hippocampus, and the right hippocampus.

**Table 2:** Volumetric measurements (mean and standard deviation in mm^3^) and group comparison results (volume group differences and P values) of the left and right amygdala and hippocampus.

	HC Volumes	SZ Volumes	Volume Group Differences		Volume P Values	Shape P Values
Left amygdala	1712.03 ± 272.03	1594.89 ± 285.94	−0.3206	117.14	<0.0001	<0.0001
Right amygdala	1957.90 ± 309.94	1793.40 ± 296.57	−0.3093	164.5	<0.0001	<0.0001
Left hippocampus	3401.41 ± 469.76	3240.85 ± 501.91	−0.3116	160.56	0.0116	0.0295
Right hippocampus	3490.02 ± 509.15	3350.55 ± 551.91	−0.2532	139.47	0.0229	<0.0001

The first column under “Volume Group Differences” denotes the z-score and the corresponding second column denotes the absolute volume difference in mm^3^.

### Shape Comparison

**Figure 1:**
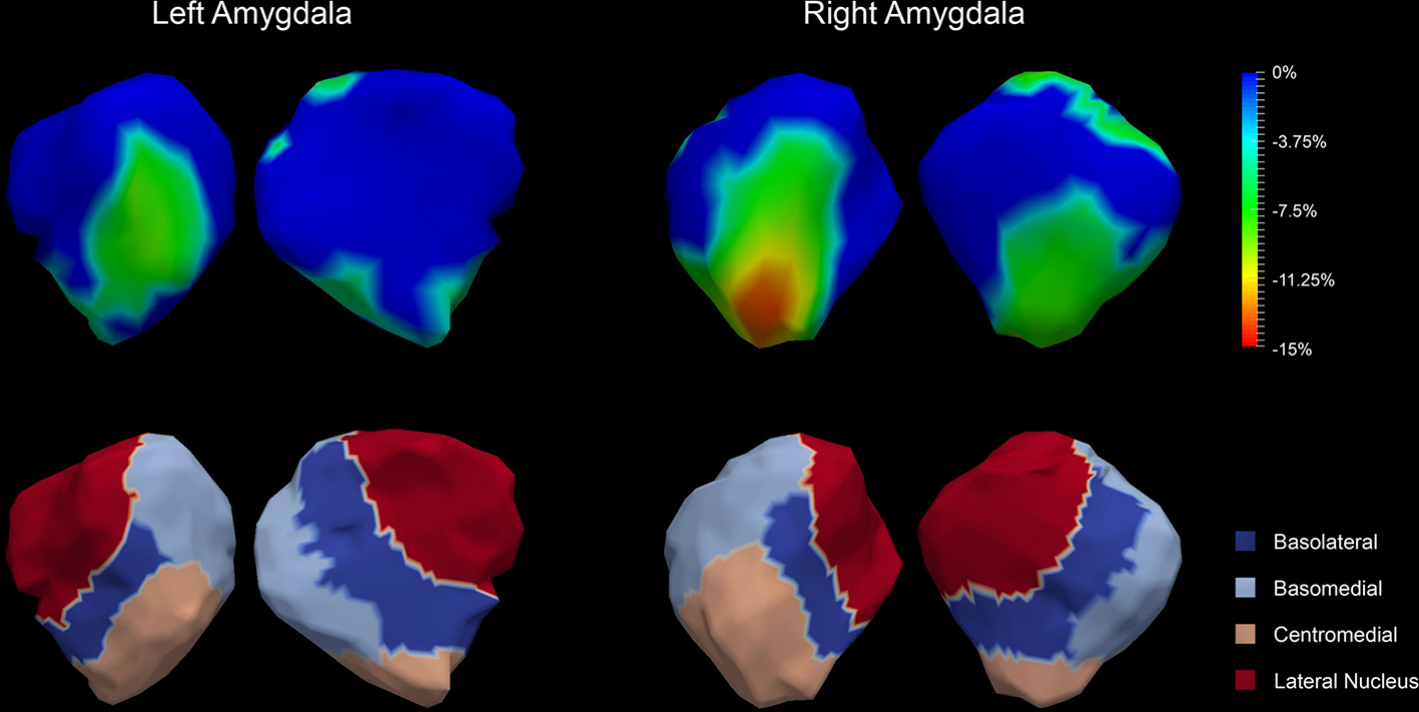
Shape analysis results for the bilateral amygdalas. Statistically significant group comparison results for the amygdalar shape in each hemisphere as well as the corresponding subregion definitions. The color bar represents the percentage of atrophy at a specific vertex in first-episode schizophrenia (FES) relative to healthy control (HC). The bottom panel illustrates the four subregions of the bilateral amygdalas. Two views (left: lateral, right: medial) are presented for each case.

**Figure 2:**
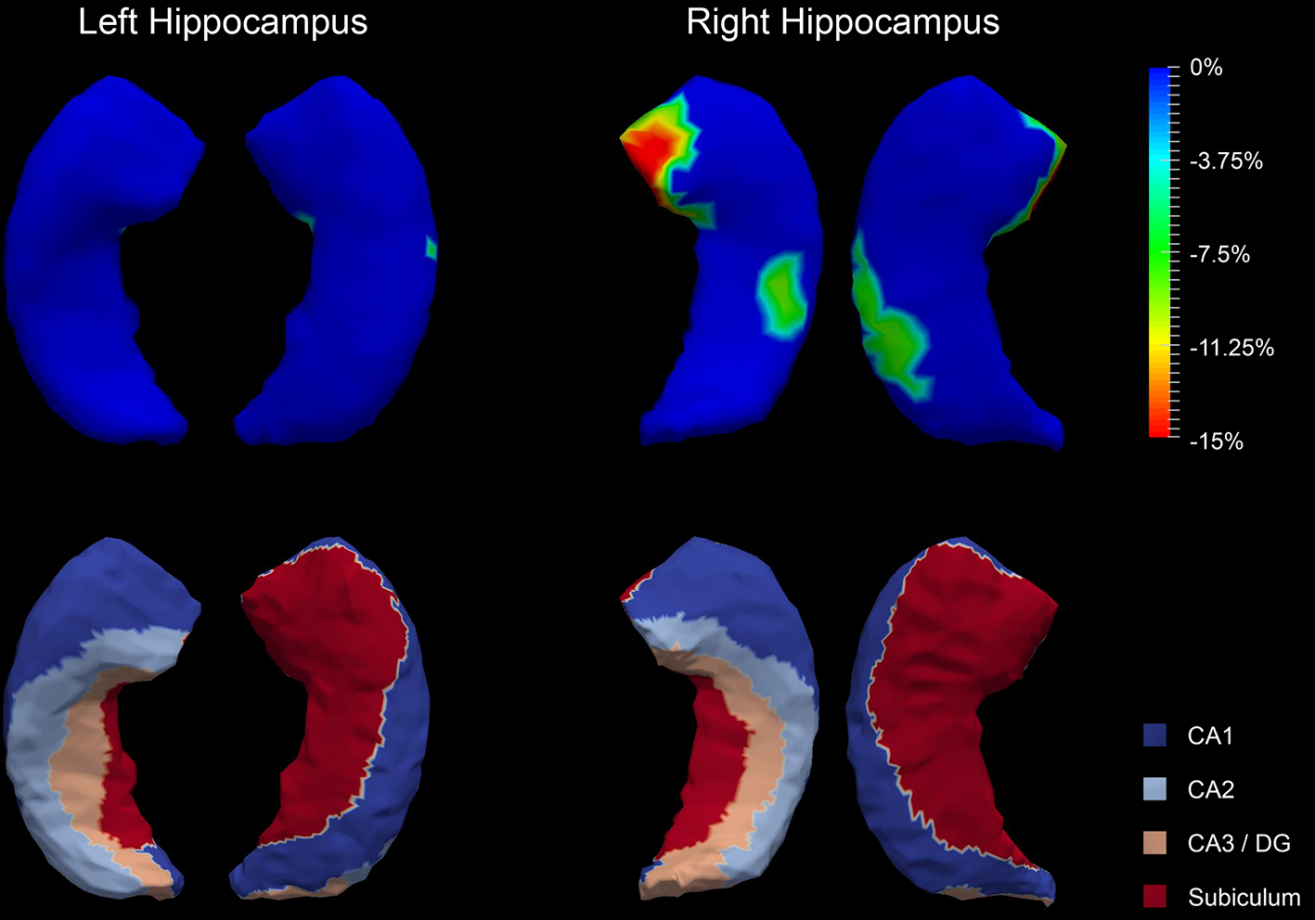
Shape analysis results for the bilateral hippocampi. Statistically significant group comparison results for the hippocampal shape in each hemisphere as well as the corresponding subregion definitions. The color bar represents the percentage of atrophy at a specific vertex in first-episode schizophrenia (FES) relative to healthy control (HC). The bottom panel illustrates the four subregions of the bilateral hippocampi. Two views (left: lateral, right: medial) are presented for each case.

The P values obtained from HC-versus-FES shape group comparisons are listed in **Table 2** as well. Significant shape group differences between FES and HC have been observed for all four structures of interest. The significance of the amygdalar shape differences was again stronger than that of the hippocampal ones (left amygdala: P < 0.0001, right amygdala: P < 0.0001, left hippocampus: P = 0.0295, right hippocampus: P < 0.0001), being consistent with the aforementioned volume findings. The shape difference mappings of the bilateral amygdalas and the bilateral hippocampi are respectively demonstrated in **Figures 1** and **2**, wherein only vertices surviving the FWER multiple comparison correction are highlighted. For vertices exhibiting significant group difference, the mean and standard deviations of the effect size (as measured by Cohen's d) are respective 0.512 ± 0.081, 0.572 ± 0.140, 0.493 ± 0.013, and 0.619 ± 0.126 for the left amygdala, the right amygdala, the left hippocampus, and the right hippocampus. Significant region-specific inhomogeneous surface atrophies were detected in the FES group for each of the four structures of interest. The corresponding surface subregion divisions are also shown in those two figures. Quantifications of those shape findings are summarized in **Table 3**. To be specific, we computed the percentage of the surface area of vertices exhibiting significant HC-versus-FES group difference for the entire structure and also each single subregion. As shown in **Figure 1** and **Table 3**, for the amygdala, the FES-related localized surface atrophies mainly occurred at the basolateral and centromedial subregions and the strongest surface atrophies occurred at the centromedial subregion. For the hippocampus, as revealed in **Figure 2** and **Table 3**, the localized surface atrophies mainly occurred at the CA1 and CA2 subregions and those two subregions also had the strongest hippocampal atrophies. For the same structure at different hemispheres, the one on the right hemisphere was affected more in terms of both atrophy degree and atrophy amount, especially for the hippocampus.

**Table 3:** Percentage of the surface area of vertices exhibiting significant FES-related atrophies for the entire structure and also each single subregion of the left and right amygdala and hippocampus.

	**Left Amygdala**	**Right Amygdala**
Entire structure	21.53%	41.10%
Basolateral subregion	30.89%	55.14%
Basomedial subregion	16.23%	28.13%
Centromedial subregion	62.87%	96.12%
Lateral nucleus subregion	1.30%	16.17%
	**Left Hippocampus**	**Right Hippocampus**
Entire structure	0.53%	13.41%
CA1 subregion	0.04%	18.59%
CA2 subregion	0.00%	15.71%
CA3/DG subregion	0.00%	14.78%
Subiculum subregion	1.25%	6.65%

## Discussion

In this study, we investigated the amygdalar and hippocampal morphometric abnormalities in FES in terms of both volume and shape. A sophisticated deformation based statistical shape analysis pipeline has been employed to quantify the localized morphometric abnormalities of the left and right amygdala and hippocampus. Several findings on FES have emerged from this study: (1) significant global volume reductions and region-specific localized surface atrophies were detected in each of the four structures of interest. (2) The statistical significance of the amygdala, in terms of both volume and shape abnormalities, was much stronger than that of the hippocampus. (3) For the same structure, the one on the right hemisphere was affected more by the FES pathology, in terms of both atrophy degree and atrophy amount, especially for the hippocampus. (4) The basolateral and centromedial subregions of the amygdala and the CA1 and CA2 subregions of the hippocampus were affected the most compared to other subregions.

One highlight that is worth being pointed out is that this study included a relatively larger number of participants (a total of 198) compared to other FES shape studies focusing on the amygdala or the hippocampus. For example, Narr et al. (11) has analyzed 122 participants and Qiu et al. (13) has included only 56 participants. A relatively large sample size ensures the statistical power of this study and the robustness of the observed findings.

In our study, we found significant amygdalar and hippocampal atrophies in FES, either globally or locally. Those observed abnormalities in FES generally agree well with previous findings (3, 5, 9), and confirm the presence of such structural abnormalities at the onset of schizophrenia which manifest in chronic patients as well (36, 37). These findings to some degree confirm the presence of amygdalar and hippocampal atrophies at an early phase in the pathology of schizophrenia. The amygdala plays a crucial role in emotional learning, and the hippocampus has been implicated to mediate encoding and retrieval of multi-modal sensory information. Therefore, the observed morphometric alterations in those structures in FES may be considered as key factors in the deficits of episodic memory and emotional learning in schizophrenia, which is also keeping in line with general hypotheses (38, 39).

Previous schizophrenia-related amygdalar and hippocampal morphometric studies have mainly focused on the global volume measurement but not the localized shape morphometrics. There have been several studies investigating the shape phenotype of the hippocampus in schizophrenia but not FES, mainly utilizing shape based deformations as well (14, 15). However, the amygdalar shape analyses are very rare in schizophrenia, especially in FES, with Qiu et al. (13) being the only such one to the best of our knowledge. This study is the first of its kind to have conducted statistical shape analysis of the hippocampus as well as the amygdala in a large sample size dataset. This is also the first FES study to have analyzed subregional shape abnormalities in the amygdala and the hippocampus.

Findings from this study suggest that the morphometric abnormalities of the amygdala are stronger and more significant than those of the hippocampus in FES. Similar abnormality patterns have also been reported in a previously-published work investigating the amygdalar and hippocampal volume and shape characteristics in FES as well (13). In that study, significant volume reductions were identified in FES for the bilateral amygdalas but not for the bilateral hippocampi. That study also reported region-specific atrophies on almost everywhere of the bilateral amygdalas, but the hippocampal shapes were only slighted affected. Similar amygdala being affected more than hippocampus observations have also been reported in another schizophrenia shape study (40). Evidence suggests that there is decreased amygdala corticotropin-releasing factor binding protein in schizophrenia (41), which may have made the amygdala be more vulnerable to the pathology of schizophrenia than the hippocampus. Also, the amygdala is of particular importance for the induction of abnormal circuitry in schizophrenia, and its growth during late adolescence and early adulthood may help to reveal FES pathogenesis (42).

There have been evidences suggesting that the somatostatin (SST) and neuropeptide-Y (NPY) expression in the amygdalar neurons plays a key role in the regulations of fear and stress responses as well as anxiety which manifest over the entire trajectory of schizophrenia (43). Some studies have also shown that SST and NPY have significant anti-anxiety effects, with a prominent involvement of the amygdalar circuitry (44, 45). As the emotional condition worsens, atrophy of the amygdala starts at one side and then gradually shrinks to the other side in the pathology of schizophrenia. There is also evidence of a significant increase of dopamine in the right amygdala in schizophrenia over healthy population (46). It is likely that the right amygdala is more relevant to the mood in schizophrenia than the left one, especially in FES. This may explain our amygdalar right>left patterns in terms of the global volume reductions and local shape atrophies. There has also been evidence suggesting that the subiculum volume of the right hippocampus was significantly associated with the verbal memory function (47). Thus, we speculate that the subiculum volume of the right hippocampus is more susceptible to the schizophrenia pathology than the left side, because it has a stronger relationship with memory. This also, to some extent, supports our hippocampal right>left observation.

The observed FES-related amygdalar atrophies were found to concentrate on the basolateral and centromedial subregions. Studies have already shown that the basolateral amygdala plays a key role in the abnormal behavior of schizophrenia (48). The reasons are potentially two-fold: firstly, one of the most important brain regions in schizophrenia, the prefrontal cortex, receives heavy inputs from the basolateral amygdala. Secondly, activity of the basolateral amygdala is selective for differently valued outcomes and is modulated by errors in reward prediction (49), which correlates to deficits in schizophrenia. The centromedial amygdala is largely involved in the execution of fear responses and its deactivation may result in impairments in fear expression and acquisition (50). The centromedial amygdala has been found to exhibit reduced activity to positively valenced faces in schizophrenia (51).

The hippocampus has been verified to be associated with schizophrenia and a variety of studies have repeatedly proven hippocampal changes in schizophrenia in terms of both structure and function. Reduced volumes, abnormal *in vivo* functions, and replicable molecular pathologies of the hippocampus have been reliably identified across different schizophrenia studies (52, 53). Declarative memory, which is known to depend on the combined memory function of the hippocampus, is one of the most persistently impaired cognitive functions in schizophrenia (54). In vivo biomarkers of hippocampal dysfunction in schizophrenia are found to be associated with the psychotic symptom characteristics in schizophrenia patients without medication (55).

The hippocampal subregions have different and continuous functions in declarative memory formation and are affected differently in the neuropathology of psychiatric diseases (56). In this study, we found the strongest FES pathology effect in the CA1 and CA2 subregions of the hippocampus. This agrees well with the subregional findings obtained in a previous FES-related hippocampal shape analysis wherein the hippocampal shape abnormalities were detected in the anterior and midbody CA1 and CA2 regions in FES patients (11). Schobel et al. reported increased metabolism in the CA1 and CA2 subregions in absence of structural differences at pre-psychotic stages, and the hippocampal volume decreased when patients progressed to a psychotic syndrome, which may indicate that CA1 and CA2 are crucial subregions of the hippocampus in FES (57).

This study still has limitations. Firstly, this study was cross-sectional. To better understand the pathology effects of FES exerted to the amygdalar and hippocampal morphometry, longitudinal studies are needed, which will be one of our future research endeavors. Secondly, the reported right>left shape differences were not obtained from rigorous statistical quantifications but instead qualitative assessments. It would be desirable to look at laterality indices to quantify the inter-hemisphere differences. However, given that the shape of the same structure at the two different hemispheres was parameterized differently, such laterality indices were infeasible at a vertex level. In other words, there is no correspondence between the surface vertices of the same structure at different hemispheres or the total number of vertices may even be different. A future research direction is to resolve this issue relying on advanced manifold transformation techniques. Lastly, more subtypes of schizophrenia, such as chronic schizophrenia, should have been included and analyzed to better understand the evolution mechanism of schizophrenia.

## Data Availability Statement

The datasets generated for this study are available on request to the corresponding author.

## Ethics Statement

The studies involving human participants were reviewed and approved by The First Affiliated Hospital of Shenzhen University Ethics Committee. The patients/participants provided their written informed consent to participate in this study.

## Author Contributions

Author XT designed the entire study and the analysis pipeline. Authors MC and WH performed all experiments and associated statistical analyses. Author GL collected MRI data and wrote the first draft of the manuscript. Author YL managed participant involvement. All authors contributed to the article and approved the submitted version.

## Funding

This work was supported by the Shenzhen Basic Research Program (JCYJ20190809120205578), the National Key R&D Program of China (2017YFC0112404), the High-level University Fund (G02236002), and the National Natural Science Foundation of China (81501546).

## Conflict of Interest

The authors declare that the research was conducted in the absence of any commercial or financial relationships that could be construed as a potential conflict of interest.
